# Questionable advisability of vitamin and mineral dietary supplement use in adolescents

**DOI:** 10.1186/s40795-023-00768-z

**Published:** 2023-09-28

**Authors:** Katja Zdešar Kotnik, Barbara Koroušić Seljak, Matej Gregorič, Gregor Jurak, Petra Golja

**Affiliations:** 1 Biotechnical Faculty, Department of Biology, University of Ljubljana, Vecna pot 111, Ljubljana, SI-1000 Slovenia; 2 Computer Systems Department, Jožef Stefan Institute, Ljubljana, Slovenia; 3 National Institute of Public Health, Ljubljana, Slovenia; 4 Faculty of Sport, University of Ljubljana, Ljubljana, Slovenia

**Keywords:** Micronutrients, Dietary intake, Dietary supplement, Food group

## Abstract

**Objective:**

Present study aimed to assess potential health risk in Slovenian adolescents due to inadequate diet and/or dietary supplement (DS) use.

**Methods:**

Data on DS use, micronutrient intake (24-h recall), eating habits (FFQ), body height and mass were collected within ACDSi (Analysis of Children’s Development in Slovenia) cross-sectional study conducted in 2014. Adolescents enrolled in first year of 15 secondary schools (average (SD) age: 15.4 (0.7) years, N = 342) were included in the sample.

**Results:**

Adolescents’ use of DS (especially multivitamins ingested as a popular drink (60%), magnesium (16%), and vitamin C (10%)) significantly contributed to their absolute intake of vitamins/minerals, resulting in higher percentage of DS users meeting reference values proposed by the nutrition societies of Germany, Austria, and Switzerland (D-A-CH recommendation). Simultaneously, DS users did not exceed the upper tolerable level proposed by the European Food Safety Authority for daily intake. With diet alone, adolescents consumed less than recommended amounts of the following vitamins/minerals: the intake was lowest for fat-soluble vitamins A, D, and E; water-soluble vitamins folate, biotin, and pantothenic acid; and minerals fluoride, iodine, chromium, and molybdenum. Suboptimal intake was due to the fact that around ¾ of adolescents consumed less than 54% of the recommended amounts (according to Optimized Mixed Diet (OMD) recommendations) for fruits, vegetables, milk/dairy products, fish, and cereals/cereal products. In contrast, the diet contributed to the consumption of 200–300% of D-A-CH minimum value for sodium. Furthermore, almost ¾ of adolescents exceeded the recommended amount for meat/meat products (320% of OMD) and sweet/salty snacks (453% of OMD).

**Conclusions:**

Although DS use improved micronutrient intake in adolescents (especially vitamin C and magnesium), activities on public-health interventions should be focus to improve their diets, especially to achieve increased intakes of fruits, vegetables, cereals/cereal products and milk/dairy products, and to reduce consumption of sweet/salty snacks and meat products.

## Introduction

Varied and balanced diet, together with adequate energy intake allowing the maintenance of body mass, is the best source of all necessary nutrients. Therefore, the use of dietary supplementation in healthy people (with vitamin D supplementation as the only exception in omnivores [1]) is neither endorsed nor recommended [2]. As adolescence is a period of rapid growth and development that increases the need for nutrients, it is crucial to provide adequate energy and nutrient intake to maintain health and prevent chronic diet-related non-communicable diseases in adulthood [3].

Studies demonstrate that most adolescents do not meet the recommended daily portions of preferred food groups such as fruits, vegetables, cereals, fish, and vegetable oils [2, 4–6]. Malnutrition related to certain micronutrients (most commonly folate, vitamin D, calcium, and iron) is therefore a common issue in this population in developed countries [7–9], despite a generally large availability and diversity of food, and thus a high possibility for maintaining a high-quality diet.

In addition to food intake, the use of dietary supplement (DS) is widespread. In adolescent, especially the use of vitamins, minerals, and their combinations [10]. In case of an unbalanced diet and low nutrient intake, DS can help users to achieve the recommended levels for certain nutrients and may have beneficial effects on health [11, 12]. In contrast, reckless use of DS can lead to toxic effects and increase short- and/or long-term negative health outcomes [13, 14]. Namely, some studies revealed regular DS users are also more likely to have healthier dietary habits than non-users [15], which likely limits their need for DS use and increases the risk of excessive nutrient intake. However, not all studies confirmed that [16]. As an exception, supplementation with vitamin D has been recommended to healthy adults, when its adequate daily amounts are not provided with UV-B light-induced biosynthesis and/or dietary intake [1].

Although several studies have examined either the overall diet of general population of adolescents [2, 4–6, 17] or their micronutrient intake from food and/or DS [18, 19], there have been few studies in adolescents so far examining these factors together [20, 9]). To our knowledge, no study in Slovenia has so far examined micronutrient intake from all sources (food + DS) in relation to different food groups consumption.

Therefore, the aim of the present study was to determine whether there exists a health risk in Slovenian adolescents due to inadequate diet and/or use of DS. For this reason, we assessed the micronutrient intake in DS users and non-users (from food and from food + DS), as well as the percentage of adolescents meeting the estimated average requirement (EAR) or the adequate intake (AI) of micronutrients.

## Subjects and methods

### Study design and study sample

Data were collected within a wider ACDSi 2014 (Analysis of Children’s Development in Slovenia) cross-sectional study [21] between September 8 and December 22, 2014. The study protocol was approved by the Ethics Committee of the Republic of Slovenia (52/03/14 and Appendix 66/11/12). The sample was selected using a two-stage procedure with clustered and stratified sampling. In the first stage, 15 schools were selected from 170 based on educational program and location. Then, a certain number of classes from each school were randomly selected. Students in these classes were then invited to voluntarily participate in the study, and written informed consent was obtained. The goal was to achieve national representativeness, requiring a sample of 384 students from the entire 2014 secondary-school population of 75,325, with a 95% confidence interval. A detailed description of the overall ACDSi 2014 study design is available elsewhere [21].

The pupils, who were enrolled in the first-year of secondary school, were presented with an additional section on nutrition in the questionnaire. Therefore, this subsample was included in the analysis of the present study. There were no additional inclusion/exclusion criteria regarding different chronic diseases or allergies.

### Dietary intake assessment

Daily nutrient intake from food was assessed using 24-hour recall method (24-h recall) as proposed by the European Food Safety Authority (EFSA) [22]. Twelve different trained interviewers conducted 24-h recall of food intake, which was repeated twice with the same participants, two weeks to one month apart. Portion sizes were estimated using the national picture book [23]. Food intake data were entered into the Slovenian web-based dietary assessment tool Open platform for clinical nutrition [24], which is validated for nutrient assessment [25]. All the entries were double checked by 2 trained interviewers. We then considered the average dietary intakes calculated by the OPEN tool from the two 24-hour recalls, which was only feasible for those individuals who completed both 24-hour recalls.

Daily intake of food groups (fruits, vegetables, cereals and cereal products, pasta/rice/potatoes, milk and dairy products, meat/meat products, fish, eggs, sweet and salty snacks, beverages) was assessed using Healthy Nutrition Score for Kids and Youth - HuSKY [26], based on data obtained using the Food Frequency Questionnaire (FFQ) adapted from KiGGS [27]. The HuSKY model is based on a comparison of reported amounts of food intake with the recommendations for Optimized Mixed Diet (OMD) [28].

### Dietary supplement assessment

Participants who had used any DS in the past 12 months were asked to complete a guided, custom designed e-questionnaire about their DS use. We collected detailed information about DS (brand name, the name of the specific product(s), the technological form of the product(s)). In addition, information about the frequency of DS use in the past year (1–3 times/year, 4–11 times/year, 1–3 times/month, 1–3 times/week, 4–6 times/week, every day, more than once a day) was collected for each specified product. Micronutrient intake from all reported DS was calculated for each participant using the reported frequency of consumption in 12 months and nutrient composition per standard serving of a particular DS. If a person used a particular product of DS less frequently than once a day, the calculation of their nutrient intake from this product was adjusted to reflect their average daily intake. Composition data for each DS were obtained from the Slovenian label-based database P3 Professional [29]. To standardize the units for each micronutrient between specific products, standard conversions were used. If there was insufficient data regarding brand, name, or frequency of use, data were excluded from further analysis (N = 18 adolescents).

### Statistical analysis

The micronutrient intake data were adjusted for energy intake using the residual method as proposed by Willet and Stampfer [30]. Namely, energy-adjusted micronutrient intake was calculated using a linear regression model, with total energy intake as the independent variable and absolute micronutrient intake as the dependent variable. The data were previously logarithmically transformed as recommended for the residual method [30]. The predictive values for each vitamin/mineral adjusted for the individual’s estimated energy intake were then obtained.

Because distributions of intake data for each vitamin/mineral were asymmetrical (defined according to the normal Q-Q plot, skewness, kurtosis, and test of normality), statistical analyses were performed using the appropriate nonparametric tests.

To examined whether the use of DS significantly contributes to the (additional) intake of vitamins/minerals in adolescents, and to what extent the additional intake with DS might exceed the tolerable upper limit of daily intake (UL), we made several comparisons.

First, vitamin/mineral intake from food in DS users was compared with their total vitamin/mineral intake (from food + DS). The comparison was performed using the nonparametric Wilcoxon test of equivalent pairs (for two dependent samples). In addition, we compared vitamin/mineral intake from food only between DS users and non-users, as well as vitamin/mineral intake from food only in non-users with vitamin/mineral intake from food + DS in users, in both cases using the Mann Whitney U-test (for two independent samples).

Second, the total daily vitamin/mineral intake (from food + DS) among DS users was compared with the UL [31] for the adolescent age group, separately by gender. The comparison was performed for the vitamins/minerals for which this limit has been established (i.e. vitamin A, D, E, niacin, B6, folate, biotin, Ca, Mg, Fe, Zn, Cu, K, Mn, Na). A non-parametric Wilcoxon test of predicted ranks for one sample was applied for his purpose.

We compared the percentages of adolescents who did not meet and met the D-A-CH recommended levels for each vitamin/mineral (determined for the adolescent age group 15–18 years, separated by gender. We conducted comparisons: a) between non-users (food intake) and users (food intake) (Chi-squared independence test); b) between non-users (food intake) and users (food + DS) (Chi-squared independence test); c) between users (food intake) and users (food + DS) (McNemer test).

To assess adolescents ' overall diets, their reported absolute amounts of food groups consumed were compared with OMD recommendations [28] using the nonparametric Wilcox test for one sample. Differences in intake of specific food groups between DS users and non-users were assessed using a Mann-Whitney U-test.

All data were analyzed using the SPSS statistical program (IBM SPSS Statistics 22, 2013). The level of statistical significance was set at 0.05.

## Results

Data are presented as average (standard deviation) in case of normal distribution of variables, or as median (5th − 95th percentile) in case of asymmetric distribution of variables.

The questionnaire on DS and two 24-h recalls were completed by 80% of the participating pupils of the first year of secondary school, thus, the final sample included 342 adolescents (162 males, 180 females), who were on average 15.4 (0.7) years old.

Among adolescents in our sample, the percentage of DS users was 29% for both genders. The majority of DS users (60%) consumed powdered multivitamins dissolved in water and used as a popular drink, 15% reported using magnesium DS, and 10% vitamin C. The remaining participants reported using various other multivitamins/minerals, proteins, and herbal products.

### Daily intake of vitamins/minerals and fulfillment of D-A-CH dietary recommendations in DS users and non-users

Table 1 (for males; N = 162) and Table 2 (for females; N = 180) present three different comparisons of absolute average daily intake of vitamins/minerals between users and non-users.

**Table 1:** Daily intake of vitamins/minerals from food, and from food + dietary supplements (DS) combined, in adolescent male non-users and users of DS.

		Males N = 162											
		**Non-users** N = 115					**DS Users** N = 47						
	**D-A-CH**	**Food**					**Food**		**Food + DS**				
	**(2016)**	M (5th – 95th percentil)					M (5th – 95th percentil)		M (5th – 95th percentil)			***p***	
**VITAMINS** (units/day)													
Vitamin A (µg)	1100	**469**				(322–605)	**517**	(341–732)**	**517**		(341–732) **	1.0	
Vitamin D (µg)	20	**1.4**				(0.8–2.2)	**1.7**	(0.9–2.9)**	**1.7**		(0.9–3.0)**	0.32	
Vitamin E (mg TE)	15	**9.0**				(5.4–12.6)	**10.2**	(5.9–16.3)***	**11.7**		(65–20.9)***	0.000	
Vitamin K (µg)	70	**55**				(40–69)	**60**	(42–81)**	**60**		(42–84)**	0.32	
Vitamin B1 (mg)	1.4	**1.3**				(0.8–1.8)	**1.5**	(0.8–2.4)***	**1.6**		(0.9–2.4)***	0.000	
Vitamin B2 (mg)	1.6	**1.5**				(0.9–2.1)	**1.7**	(1.0–2.8)**	**1.9**		(1.1–3.0)***	0.000	
Niacin (mg NE)	17	**25**				(15–35)	**28**	(17–44)**	**31**		(18–47)***	0.000	
Panthotenic a. (mg)	6	**4.7**				(2.9–6.5)	**5.3**	(3.1–8.3)**	**6.1**		(3.5–10.7)***	0.000	
Vitamin B6 (mg)	1.6	**1.7**				(1.0–2.3)	**1.9**	(1.1–3.0)**	**2.1**		(1.2–3.3)***	0.000	
Biotin (µg)	30–60	**28**				(18–37)	**31**	(19–46)**	**31**		(19–49)**	0.04	
Folate (µg DFE)	300	**231**				(147–313)	**259**	(157–394)**	**285**		(170–449)***	0.000	
Vitamin B12 (µg)	3.0	**3.1**				(1.7–4.6)	**3.6**	(1.9–6.1)**	**3.9**		(2.1–6.6)***	0.000	
Vitamin C (mg)	105	**91**				(58–123)	**102**	(62–155)**	**112**		(67–273)***	0.000	
**MINERALS** (units/day)													
Calcium (mg)	1200	**1307**				(1005–1560)	**1398**	(1045–1782)**	**1398**		(1045–1782)**	0.000	
Magnesium (mg)	400	**256**				(163–347)	**287**	(175–435)**	**287**		(175–435)**	0.07	
Iron (mg)	12	**14.2**				(7.8–21.4)	**16.6**	(8.5–29.1)**	**16.6**		(8.5–31.0)**	0.32	
Zinc (mg)	10	**9.1**				(5.2–13.3)	**10.5**	(5.6–17.7)**	**/**			/	
Iodine (µg)	200	**71**				(48–91)	**78**	(51–110)**	**/**			/	
Copper (mg)	1.0–1.5	**1.5**				(0.9–2.2)	**1.7**	(1.0–2.9)**	**1.8**		(1.0–2.9)**	1.0	
Selenium (µg)	70	**72**				(45–98)	**81**	(48–124)**	**/**			/	
Potassium (mg)	2000	**2872**				(1737–4036)	**3270**	(1873–5211)**	**3270**		(1873–5211)**	0.18	
Phosphorous (mg)	1250	**1189**				(702–1698)	**1362**	(759–2220)**	**/**			/	
Fluoride (mg)	/	**182**				(107–262)	**209**	(115–344)**	**/**			/	
Sodium (mg)	550	**2581**				(1416–3871)	**3012**	(1550–5252)**	**3012**		(1550–5252)**	0.18	
Manganese (mg)	2.5–5.0	**4.2**				(2.5–6.0)	**4.8**	(2.7–7.9)**	**4.8**		(2.7–7.9)**	1.0	
Chrome (µg)	30–100	**22**				(13–32)	**26**	(14–43)**	**/**			/	
Molybdenum (µg)	50–100	**32**				(19–45)	**36**	(21–58)**	**/**			/	
Chlorine (mg)	/	**4180**				(2303–6254)	**4875**	(2519–8469)**	**/**			/	

*p* – exact p-value calculated with nonparametric Wilcoxon signed-rank test for vitamin/mineral intake in DS users, comparing intake with food and with food + DS.

*p < 0.05; ** *p* < 0.01; ***p < 0.001 – *p*-value calculated with Mann Whitney U-test for the intake of vitamins/minerals **with food** in non-users and vitamin/mineral intake **with food + DS** in users

* *p* < 0.05; **p < 0.01; ***p < 0.001 – p-value calculated with Mann Whitney U- test for the intake of vitamins/minerals **with food** in non-users and intake of vitamins/minerals **with food** in users

/ Participants did not consume this micronutrient with DS

**Table 2:** Daily intake of vitamins/minerals from food, and from food + dietary supplements (DS) combined, in adolescent female non-users and users of DS.

							Females N = 180						
							**DS Non-users** N = 128		**DS Users** N = 52				
			**D-A-CH**				**Food**		**Food**		**Food + DS**		
			**(2016)**				M (5th – 95th percentil)		M (5th – 95th percentil)		M (5th – 95th percentil)		***p***
**VITAMINS** (units/day)													
Vitamin A (µg)			900				**377**	(269–553)	**375**	(263–499)	**375**	(263–525)	0.32
Vitamin D (µg)			20				**1.0**	(0.6–1.9)	**1.0**	(0.6–1.6)	**1.0**	(0.6–3.3)	0.18
Vitamin E (mg TE)			12				**6.7**	(4.3–11.2)	**6.7**	(4.1–9.7)	**7.4**	(4.7–12.8)*	0.000
Vitamin K (µg)			60				**45**	(34–64)	**45**	(33–58)	**45**	(33–58)	0.32
Vitamin B1 (mg)			1.1				**0.9**	(0.6–1.6)	**0.9**	(0.6–1.4)	**1.1**	0.6–2.2)	0.000
Vitamin B2 (mg)			1.2				**1.1**	(0.7–1.9)	**1.1**	(0.7–1.6)	**1.3**	(0.7–2.2)	0.000
Niacin (mg NE)			13				**19**	(12–31)	**19**	(12–27)	**20**	(12–31)	0.000
Panthotenic a. (mg)			6				**3.6**	(2.3–5.8)	**3.5**	(2.3–5.1)	**4.0**	(2.4–7.5)*	0.000
Vitamin B6 (mg)			1.2				**1.2**	(0.8–2.1)	**1.2**	(0.8–1.8)	**1.4**	(0.8–2.3)	0.000
Biotin (µg)			30–60				**22**	(15–33)	**22**	(15–30)	**22**	(15–30)	0.04
Folate (µg DFE)			300				**177**	(118–281)	**176**	(115–248)	**187**	(116–294)	0.000
Vitamin B12 (µg)			3.0				**2.2**	(1.3–4.0)	**2.2**	(1.3–3.4)	**2.3**	(1.3–4.1)	0.000
Vitamin C (mg)			90				**70**	(47–111)	**70**	(46–98)	**81**	(46–145)*	0.000
**MINERALS** (units/day)													
Calcium (mg)			1200			**1121**		(886–1466)	**1118**	(872–1364)	**1118**	(872–1388)	0.000
Magnesium (mg)			350			**197**		(132–312)	**196**	(128–275)	**198**	(131–293)	0.01
Iron (mg)			15			**10.0**		(5.8–18.5)	**10.0**	(5.6–15.7)	**10.0**	(5.5–15.7)	0.32
Zinc (mg)			7.0			**6.5**		(3.9–11.6)	**6.5**	(3.8–10.0)	**6.5**	(3.8–10.1)	0.29
Iodine (µg)			200			**57**		(40–83)	**56**	(39–75)	**/**		/
Copper (mg)			1.0–1.5			**1.1**		(0.7–1.9)	**1.1**	(0.7–1.7)	**1.1**	(0.7–1.7)	0.32
Selenium (µg)			60			**55**		(36–88)	**54**	(35–77)	**/**		/
Potassium (mg)			2000			**2142**		(1364–3583)	**2129**	(1322–3118)	**2129**	(1322–3118)	0.18
Phosphorous (mg)			1250			**874**		(545–1499)	**868**	(527–1296)	**/**		/
Fluoride (mg)			/			**133**		(82–231)	**132**	(80–199)	**/**		/
Sodium (mg)			550			**1819**		(1062–3359)	**1806**	(1023–2847)	**1806**	(1024–2847)	0.18
Manganese (mg)			2.5–5.0			**3.1**		(1.9–5.3)	**3.1**	(1.9–4.6)	**3.1**	(1.9–4.6)	0.32
Chrome (µg)			30–100			**16**		(10–28)	**16**	(10–24)	**/**		/
Molybdenum (µg)			50–100			**24**		(15–40)	**24**	(15–35)	**/**		/
Chlorine (mg)			/			**2953**		(1730–5431)	**2931**	(1667–4608)	**/**		/

*p* – exact p-value calculated with nonparametric Wilcoxon signed-rank test for vitamin/mineral intake in DS users, comparing intake with food and with food + DS.

* *p* < 0.05; ** *p* < 0.01; *** *p* < 0.001 – p-value calculated with Mann Whitney U-test for the intake of vitamins/minerals **with food** in non-users and vitamin/mineral intake **with food + DS** in users

* *p* < 0.05; ** *p* < 0.01; *** *p* < 0.001 – p-value calculated with Mann Whitney U- test for the intake of vitamins/minerals **with food** in non-users and intake of vitamins/minerals **with food** in users

/ Participants did not consume this micronutrient with DS

*1) Comparisons between the absolute intake of vitamins/minerals with food and with food + DS in DS users.* Results demonstrated that the use of DS significantly contributed to the (additional) intake of most vitamins (E, all B vitamins, and C) and some minerals (calcium in both genders and magnesium in females) in adolescent DS users of both genders. Consequently, DS significantly increased the percentage of male adolescents meeting recommendations for vitamin E (17%), pantothenic acid (15%), folate (23%), and vitamin C (13%); in female adolescents, DS use increased the percentage of females who met recommendations for vitamin B1 (21%), B2 (12%), B12 (13%), and vitamin C (25%) (data not presented). DS products consumed by adolescents did not contain minerals such as iodine, selenium, phosphorus, fluoride, chromium, molybdenum, and chlorine.

*2) Comparisons between absolute dietary intake of vitamins/minerals with food in non-users and with food + DS in DS users.* Our results revealed that male users consumed significantly more of all vitamins/minerals (with food + DS) than male non-users (with food only) (*p* from 0.01 to 0.000). In females, such differences were found for vitamins E, pantothenic acid, and C (*p* < 0.05), and the trend is also evident for vitamin B2 (*p* = 0.06). DS significantly increased the percentage of males who met the recommendations for vitamins K, B1, B2, pantothenic acid, folate, vitamin C, phosphorus, vitamin E, pyridoxine, biotin, B12, and for magnesium. In females, the use of DS significantly increased the percentage of adolescents who met recommendations for vitamins B1 and C (data not presented in the Table 1 or 2).

*3) Comparisons between absolute intake of vitamins/minerals with food in non-users and with food in DS users.* Results demonstrated that DS users had significantly higher absolute intakes of all studied vitamins/minerals with food only than non-users. No such differences were found in females. Consequently, significantly more male DS users met D-A-CH recommendations with food only for vitamins K, B1, B2, pantothenic acid, folate, vitamin C, and for phosphorus compared to non-users. In females, there were no significant differences in dietary vitamin/mineral intakes with food only between non-users and DS users for any vitamin or mineral (data not presented).

**Fig. 1:**
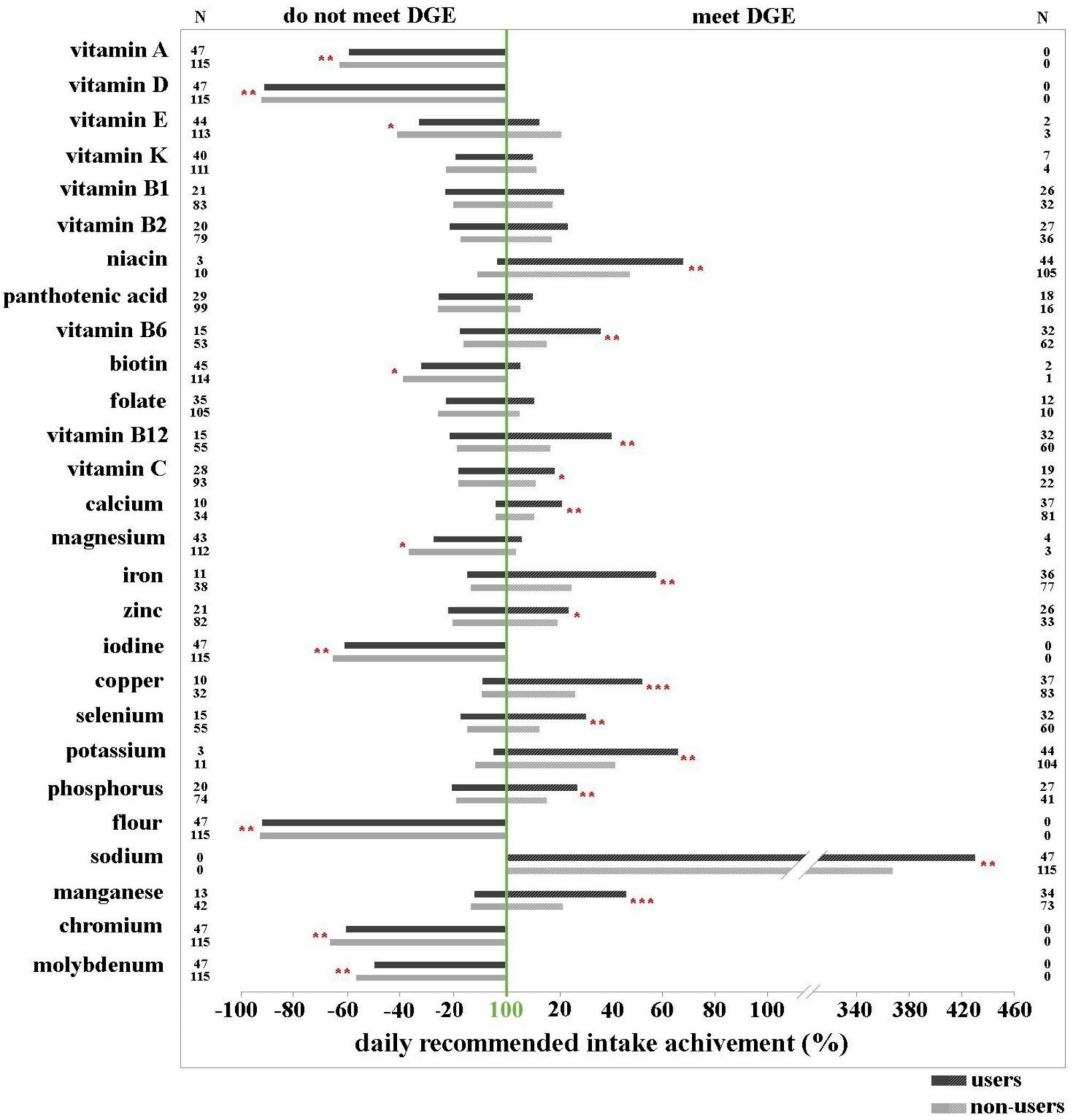
Percentage values of recommended daily intake for vitamins/minerals with diet alone according to the existing D-A-CH nutritional recommendations (D-A-CH 2016) in adolescent **males**. Data are presented separately for those who did not meet the recommendations (all columns left from the 100% line) and for those who did (all columns right from the 100% line), both for DS non-users and DS users. Significant differences in the percentages of recommended daily intakes for different vitamins/minerals between non-users and users are also presented (* p < 0.05; ** p < 0.01; *** p < 0.001; tested with Mann Whitney U-test)

**Fig. 2:**
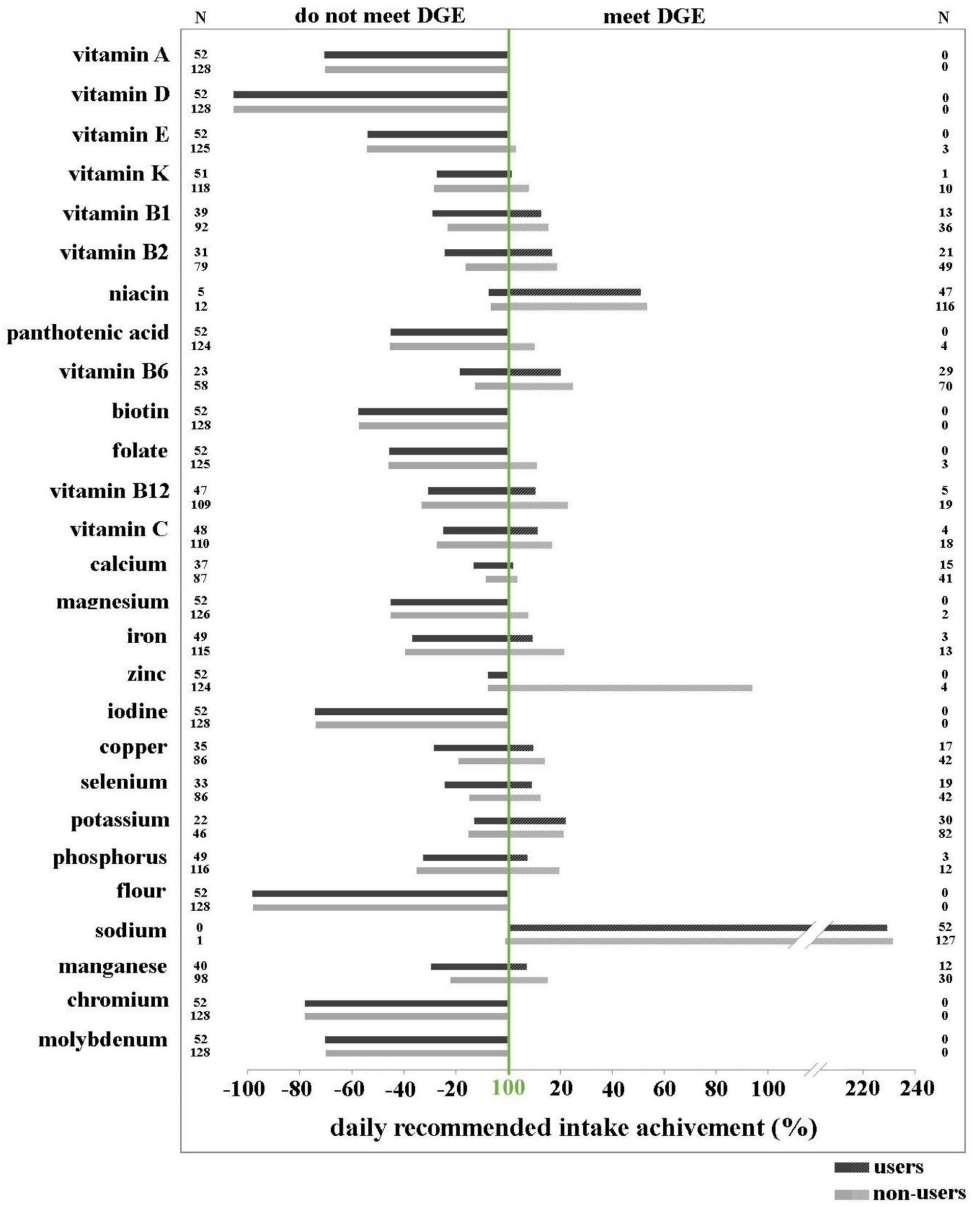
Percentage values of recommended daily intake for vitamins/minerals with diet alone according to the existing D-A-CH nutritional recommendations (D-A-CH 2016) in adolescent **females**. Data are presented separately for those who did not meet the recommendations (all columns left from the 100% line) and for those who did (all columns right from the 100% line), both for DS non-users and DS users. Significant differences in the percentages of recommended daily intakes for different vitamins/minerals between non-users and users are also presented (* p < 0.05; ** p < 0.01; *** p < 0.001; tested with Mann Whitney U-test)

Figure 1 (for males) and Fig. 2 (for females) present the differences in percentage of daily intake of vitamins/minerals relative to D-A-CH recommendations between DS users and non-users. Results demonstrated that in the group of males, who did not meet the D-A-CH recommendation, DS users achieved higher percentages of the recommended daily intake from food alone than non-users for vitamins A, D, E, biotin, and minerals magnesium, iodine, fluoride, chromium, and molybdenum. The same was true in the group of males, who met the D-A-CH recommendation for niacin, vitamin B6, B12, C, calcium, iron, zinc, copper, selenium, potassium, sodium, and manganese. No such differences were observed in females. In addition, compared to the D-A-CH recommendations, adolescents had the lowest intakes of fat-soluble vitamins A, D, and E, and some water-soluble vitamins (folate, biotin, and pantothenic acid), particularly noticeable for water-soluble vitamins in females. Among minerals, intakes were lowest for fluoride, iodine, chromium, and molybdenum in both genders. In contrast, compared to the D-A-CH recommendations, the excessive intakes were highest for niacin and sodium in both genders. Adolescents consume consumed from 200 to 300% of the estimated D-A-CH recommended minimum value for sodium. Results in Figs. 1 and 2 demonstrates that users of DS have higher intakes of vitamins and minerals through diet alone, as compared to nonusers (which is valid for both groups, thus those who meet the recommendations and those who do not). This suggests that there is an urgent need to improve dietary habits of all adolescents, rather than encourage the use of DS among adolescents: namely, there exists a group of adolescents, who already have an adequate micronutrient intake by food alone, but nevertheless use DS; and there exists a group of adolescents, who do not have an adequate micronutrient intake despite DS use.

### Exceeding the tolerable upper intake level

**Fig. 3:**
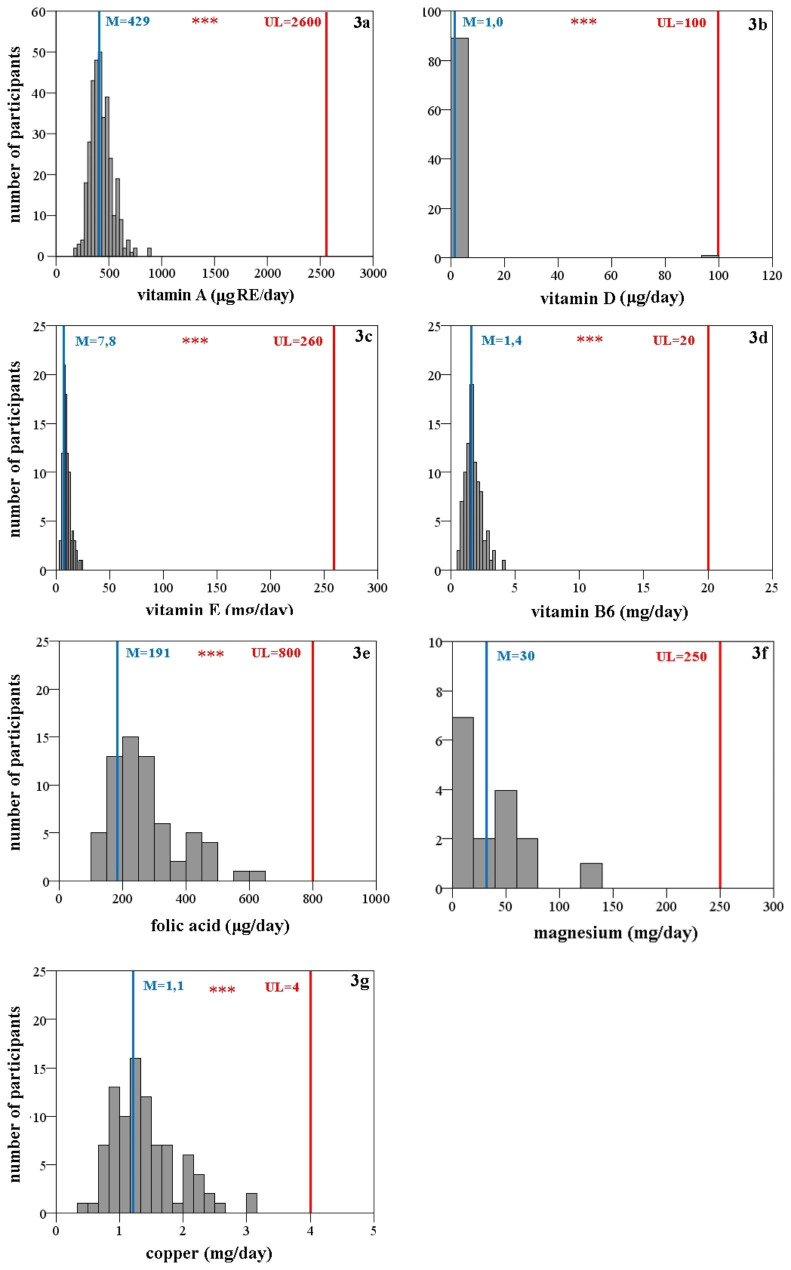
**(a – g)**: Distribution of intakes of selected vitamins/minerals from food + dietary supplements (DS) in adolescent DS users in both genders. The figure also presents the median of the sample (M; blue), the upper level intake (UL; red), and the statistical significance between the two, calculated using Wilcoxon signed rank test for one-sample (* *p* < 0.05; ** *p* < 0.01; *** *p* < 0.001)

The distribution of intakes of vitamins/minerals (Fig. 3) consumed by adolescents, from food + DS, is presented in comparison with the UL for those vitamins/minerals, for which this limit has been set by EFSA [31]. For magnesium, only intake from DS was considered, as the UL is set for the intake from DS only. The distribution of niacin intake is not presented, because no information on the form of niacin intake (i.e. nicotinamide or nicotinic acid) has been available. Results demonstrate that adolescentsˈ use of DS did not contribute to such an increased intake of vitamins/minerals to exceed the upper limit.

### Assessment of the intake of specific food groups

Table 3 presents the absolute intake of specific food groups by gender in DS users and non-users and comparisons to the OMD recommendations. Results demonstrated that male DS users consumed significantly more fruit, milk/dairy products, and tended to consume more vegetables (*p* = 0.06) than male non-users. In addition, male DS users did not meet the OMD recommendation for pasta/rice/potatoes (*p* = 0.000), and the same trend could also be observed for vegetables (*p* = 0.05). For male non-users the same was true for vegetable, fruits, milk/dairy products, and beverages (*p* = 0.000 for all groups) and a similar trend was also observed for cereals/cereal products (*p* = 0.05). In contrast, a highly significant excess of OMD recommendation for meat/meat products and sweet/salty snacks was observed in both male DS users and non-users. Female DS users consumed significantly more vegetables (*p* = 0.05) and tended to consume less meat/meat products (*p* = 0.08) than non-users. In addition, both female DS users and non-users did not meet the OMD recommendations for vegetables, fruits, cereals/cereal products, pasta/rice/potatoes, milk/dairy products, eggs, fish, and beverages. In contrast, both groups exceed the consumption of sweet/salty snacks, while non-users (but not users) also exceed the consumption of meat/meat products.

**Table 3:** Daily intake of reported absolute amounts of food groups consumed and OMD recommendations (Kersting et al. 2005), presented by gender, separately for adolescent DS users and non-users

		Males N = 162						
		Non-users N = 108			DS Users N = 44			
**FOOD GROUPS** (g/day)	**OMD** **(2005)**	M (5th – 95th percentil)		**p**	M (5th – 95th percentil)		**p**	**p**_**1**_
Vegetables	350	**148**	0–550	0.000	**199**	0–1023	0.05	0.06
Fruits	350	**119**	2–450	0.000	**124**	1–1350	0.12	0.03
Cereals/products	350	**275**	65–1228	0.05	**320**	40–1210	0.77	0.53
Pasta/rice/potatoes	350	**151**	29–586	0.000	**205**	19–598	0.000	0.12
Milk/products	500	**241**	37–1273	0.000	**339**	55–1590	0.50	0.04
Meat/products	85	**168**	39–633	0.000	**229**	21–1386	0.000	0.13
Eggs (g/week)	140	**98**	0–634	0.75	**147**	0–1838	0.26	0.10
Fish (g/week)	100	**74**	0–451	0.78	**74**	0–1278	0.99	0.90
Sweet/salty snacks	95	**194**	38–801	0.000	**260**	38–1694	0.000	0.13
Beverages	1500	**929**	326–2885	0.000	**1355**	210–3578	0.10	0.18
			**Females N = 180**					
		**Non-users** N = 121			**DS Users** N = 49			
**FOOD GROUPS** (g/day)	**OMD** **(2005)**	M (5th – 95th percentil)		**p**	M (5th – 95th percentil)		**p**	**p**_**1**_
Vegetables	300	**126**	5–529	0.000	**149**	20–965	0.02	0.05
Fruits	300	**119**	6–900	0.000	**150**	10–900	0.01	0.51
Cereals/products	280	**193**	25–696	0.000	**165**	19–540	0.000	0.26
Pasta/rice/potatoes	300	**116**	23–323	0.000	**109**	24–457	0.000	0.85
Milk/products	450	**184**	20–802	0.000	**167**	18–835	0.000	0.64
Meat/products	75	**100**	9–301	0.000	**67**	6–282	0.60	0.08
Eggs (g/week)	140	**49**	0–518	0.002	**49**	0–501	0.000	0.37
Fish (g/week)	100	**69**	0–204	0.000	**37**	0–830	0.002	0.95
Sweet/salty snacks	75	**143**	17–1133	0.000	**140**	15–1041	0.000	0.69
Beverages^1^	1400	**763**	117–2611	0.000	**880**	100–3026	0.000	0.41

*p* – exact p-value calculated using the non-parametric Wilcoxon signed rank test for one sample for each food group intake and the OMD recommendations

*p*_1_ – exact p-value calculated using Mann-Whitney U-test for the intake of each food group between non-users and DS users

^1^ Only liquids from beverages, not liquids from food, are included in the calculation of liquid intake

Furthermore, the results demonstrated (results not presented) that both males and females, who did not consume enough vegetables (82% of males and 82% of females), met on average only 36% of the OMD recommendations for vegetable intake (*p* = 0.000). Males and females, who did not consume enough fruit (77% of males and 70% of females), met on average only 30% of the OMD recommendations for fruit intake (males 27% and females 31%). Moreover, a significant proportion of adolescents (68% of males and 89% of females) did not meet half of the recommended OMD for milk intake (about 40% in both genders). The same was true for cereals/cereal products consumption; up to 68% of males and 75% of females did not meet the OMD recommendations (they met on average 54% of the OMD recommended value). Furthermore, 78% of males and 59% of females did not meet fish intake recommendations (they met on average 32% of the OMD recommended value). In contrast, the majority of males (79%) and more than half of females (55%) consumed more meat/meat products than the OMD recommended value; males exceeded meat consumption recommendations by an average of four times (403%) and females by almost 2.5 times (236%). Similarly, three quarters of adolescents of both genders (82% of males and 71% of females) were found to consume excessive amounts of sweet and salty snacks, with consumption 3 to 4 times higher relative to OMD recommendation in both genders.

## Discussion

The study demonstrate that the participating adolescents’ use of DS contributes significantly to their absolute intakes of most vitamins and certain minerals and consequently increases the percentage of adolescents (especially males), who meet the D-A-CH recommendations for their intake. Yet, the intake of vitamins/minerals is not increased to the extent that adolescent DS users would exceed the UL. Nevertheless, the vast majority of adolescents (DS users and non-users) do not meet the D-A-CH recommendations for most vitamins/minerals, reflecting the fact that diet of the majority of adolescents deviates substantially from the OMD recommendations for healthy eating. Adolescents consume significantly less fruits, vegetables, milk/dairy products, and cereals/cereal products than recommended. In contrast, the highest excessive intakes in both genders were observed for sodium. Indeed, adolescents consumed from 200 to 300% of the estimated D-A-CH minimum value for sodium, most likely due to excessive consumption of meat/meat products (except female DS users) and sweet/salty snacks. However, results demonstrate that users of DS have higher intakes of vitamins and minerals through diet alone, as compared to nonusers (which is valid for both groups, thus those who meet the recommendations and those who do not). This suggests that there is an urgent need to improve dietary habits of all adolescents, rather than encourage the use of DS among adolescents: namely, there exists a group of adolescents, who already have an adequate micronutrient intake by food alone, but nevertheless use DS; and there exists a group of adolescents, who do not have an adequate micronutrient intake despite DS use.

**Adolescents do not achieve adequate nutrient intake through their diet**, which was clearly demonstrated in our study as well as those of several other studies [32, 33, 19, 9]. According to our study, adolescents had the lowest intakes of fat-soluble vitamins A, D, E, and, mainly in females, and some water-soluble vitamins (folate, biotin, and pantothenic acid) compared to the D-A-CH recommendations. For minerals, intakes were lowest in both genders for fluoride (relative to EFSA recommendations), iodine, chromium, and molybdenum. Studies of adolescents in other developed countries frequently report specific deficiencies in certain vitamins/minerals [32] (Spain, France, UK, North Ireland, Portugal, Germany); [33] (various countries in Europe); [19] (Australia); [34] (Greece, Germany, Belgium, France, Hungary, Italy, Sweden, Austria, Spain), [8] (US), [9] (Greece). After reviewing and summarizing nutrient deficiencies in adolescents from different studies, almost the entire spectrum of nutrients has been identified as those with inadequate intake (e.g. minerals calcium, magnesium, iron, zinc, potassium, and iodine, and vitamins D, K, E, C, folic acid, B2, B6, B12), making it difficult to highlight the most important ones. Only few studies demonstrated that adolescents have adequate dietary intake of most vitamins/minerals according to D-A-CH recommendation (data were obtained using self-administered computer-based 24-h recall) [34].

**Adolescent’s sodium intake exceeds the recommendation values.** Results of our study demonstrated that adolescents consume 200–300% of the estimated D-A-CH minimum value for sodium, which is particularly valid in males. Similarly, the study HELENA [34] found that European adolescents of both genders had 3–5 times higher sodium intake compared to the D-A-CH recommendation. López-Sobaler et al. [7] even reported that usual sodium intake exceeded the UL (relative to the American Institute of Medicine (IOM) recommendations) in a considerable percentage of Spanish adolescents. The main reasons for the assessed differences in vitamins/minerals intake in adolescents between different studies are certainly different nutrient intake assessment methods (e.g. FFQ, 24-hour recall (once or twice), dietary records or weighted food records (one to seven days), different food composition database used, and finally different micronutrient intake recommendations (e.g. EFSA, D-A-CH, IOM, and various national recommendations), which may lead to different conclusions regarding the intake relative to these recommendations. Therefore, high caution is needed when interpreting and comparing the results of different studies.

**DS users had a significantly higher intake of certain micronutrients compared to non-users.** Since the use of DS is very widespread among adolescents in our sample (69%) [10], especially the use of vitamins and minerals, and their combinations (45%), it is not surprising that DS users had a significantly higher intake of certain micronutrients compared to non-users. The results of our study revealed significant contribution of DS use to increased intakes of all vitamins (except fat-soluble vitamins A, D, and K) and calcium in both genders, and magnesium in females. This finding is also consistent with the results of several other studies [12, 19, 11] that compared micronutrient intakes between DS users and non-users in adolescents. Although we found a number of studies that addressed this issue in adolescents [35–38, 12, 39, 19, 11], it was only possible to compare our results with some of them or only for individual micronutrients, as some studies focused only on specific micronutrients, or provided results in a form that was not comparable to ours. Gallagher et al. [19] also observed significantly higher intakes of all vitamins (except vitamin D), and of the mineral’s calcium, magnesium, iron, phosphorus, and copper in male DS users, and increased intakes of vitamin A, pantothenic acid, pyridoxine, folate and vitamin C, and higher intakes of magnesium and potassium in female DS users compared to non-users. Stang et al. [36] found similar results in daily DS users, who had higher intakes of all vitamins/minerals (except zinc) than non-users. In contrast, this was not observed in adolescent users who consumed DS less frequently than daily [36]. In adolescents, a study by Dwyer et al. [37] demonstrated that use of DS contributed to more than 50% of total daily intake of vitamins C and E and to more than 20% of intake of other vitamins but not minerals.

**In our study DS increased the percentage of individuals (DS users) who met the recommendations for certain vitamins**, i.e. for vitamin E, pantothenic acid, folate, and vitamin C in males and for vitamins B1, B2, B12, and C in females, which is consistent with other similar studies. A study by Gallagher et al. [19] found that the percentage of individuals meeting recommendations for vitamins D, E, and folate increased by 20% when using DS. Use of DS significantly increased the percentage of adolescents meeting recommendations for vitamin B6, folic acid, and vitamin C [36], for vitamin D and magnesium in both genders [12] and for calcium in females [12].

Interestingly, the percentage of male (but not female) DS users who met the D-A-CH recommendation for specific vitamins (K, B1, B2, pantothenic acids, folate, and vitamin C) and minerals (zinc and phosphorus) with food alone was higher than in non-users. In a study conducted by Bailey et al. [39], the authors came to similar conclusions; although there were no differences in absolute micronutrient intake (as in our study), users largely met the recommendations for vitamins A and C as well as calcium, magnesium, and phosphorus in both genders, which was not true for non-users. Most other studies comparing vitamin/mineral dietary intake in adolescents between DS users and non-users found significant differences in vitamin/mineral intake between study groups [37, 19, 11]. For example, Kang et al. [11] demonstrated, that DS users already have a higher intake of vitamin A, B2, calcium, phosphorus, and potassium with food alone than non-users. In addition, Dwyer et al. [37] reported that DS users have a higher intake of all vitamins/minerals from food (except vitamin E and B12) than non-users.

Considering vitamins/minerals from food + DS, our results demonstrated that male DS users had higher intakes of all vitamins and most minerals, while female DS users had higher intakes of vitamin E, pantothenic acid, and vitamin C than male and female non-users, respectively. Other studies indicated that adolescent users had significantly higher intake of vitamin A [39], vitamin D [12, 39, 19], vitamin E [39, 19], thiamine [11], niacin [11], pantothenic acid [11], folate [19], vitamin C [11, 39], calcium [12, 39, 19], magnesium [12, 39, 19], and iron [39, 11] as non-users.

**In our study adolescents’ vitamin/mineral intakes did not exceed the UL, either with food alone or with food + DS.** Similarly, study of Dwyer et al. [37] demonstrated that adolescents rarely exceeded the UL for any vitamin/mineral with diet (and DS) (only 1% of individuals were reported to exceed the UL limit for niacin). In contrast Shakur et al. [12] found that the use of vitamin/mineral DS increased the percentage of those children and adolescents, who exceeded the UL for zinc, folic acid, magnesium, niacin, vitamin A, and iron when they used DS. Bailey et al. [39] reported the UL was exceeded for iron and zinc in 9% and 8% of adolescents, respectively. Kang et al. [11] estimated that the percentage of adolescents exceeding the UL was 8% for vitamin A, 4% for vitamin C, and 4% for iron.

Despite the significant contribution of DS to vitamin/mineral intake in DS users, none of the adolescents in our study met recommendations for vitamins A and D and for iodine, chromium, and molybdenum. In addition, less than a quarter of adolescents of both genders met the recommendations for vitamins E, K, biotin, magnesium, and fluoride, and for pantothenic acid, folate, B12, iron, and phosphorus among female adolescents. Gallagher et al. [19] reported that more than half of Australian adolescent males did not meet recommendations for vitamin D, and more than half of adolescent females did not meet the recommendations for vitamin D, E, and folate, and for minerals calcium and magnesium, despite using DS. Stang et al. [36] found that most adolescents of both genders in the United States have deficiencies of vitamins A and E, and of minerals calcium and zinc (and achieve less than 75% of recommended levels), despite using DS. In females, iron deficiency was also observed [36].

**Inadequate vitamin/mineral intake and issues from the perspective of adolescents’ health.** Despite the generally accepted drawbacks of the method used to obtain micronutrient intake data in our study (24-h recall), the results nevertheless allow us to highlight some of the issues from the perspective of adolescents’ health. Although calcium intake has been shown to be adequate in both genders, vitamin D and phosphorus intake should also be considered in terms of normal bone structure. Because dietary intake of vitamin D is relatively insignificant compared with endogenous synthesis, we actually have no current data on vitamin D status of adolescents; nevertheless, we can assume that it is too low for most adolescents in autumn and winter, when in moderate latitudes there are fewer sunny days. We also discovered that female’s phosphorus intake was too low. Altogether, calcium, vitamin D, and phosphorus, are the key to proper bone development, with most adulthood bone mass being formed in early puberty (between 10 and 14 years for females and between 12 and 16 years for males). Based on our findings and the results of the study of Gregorič [40] in slightly younger adolescents (in which females also had lower calcium intake than recommended), we can conclude that female adolescents are exposed to more adverse outcomes than males due to low calcium, vitamin D, and phosphorus intakes. Thus, we can conclude that girls are at increased risk for fractures and the development of osteoporosis later in life due to their diet [36, 3]. In addition, more females than males in our study are at increased risk of anemia due to low iron (as well as vitamin C) intake [41].

### Adolescents consumption of food group according to OMD recommendations

#### Fruit and vegetable

The low intakes of most vitamins/minerals in adolescents in our study are not surprising, as analysis of consumption of specific food groups revealed that most adolescents consumed significantly fewer of the recommended food groups (fruits, vegetables, milk/dairy products, cereals/cereal products, and fish) compared with the OMD recommendations [28]. Only about a quarter of adolescents meet OMD recommendations for fruit (23% males and 30% females) and even fewer for vegetables consumption (18% of males and 18% of females). More worryingly, adolescents who did not meet the recommendations for fruit and vegetable consumption met on average less than 40% of the OMD recommendations for daily fruit and vegetables intake. When fruit and vegetable consumption is evaluated from the KIGGS FFQ according to the evaluation criteria of the HuSKY model, as performed in our study, one must be attentive to some of the disadvantages of this method. For the purpose of evaluation, only freshly consumed fruit is considered in the questionnaire’s fruit set, while fresh, frozen, cooked, and canned vegetables are considered in the vegetable set. This suggests that adolescents’ fruit and vegetables consumption is underestimated; studies show that more detailed questionnaires with longer food lists generally provide higher estimates of quantitative food intake, as evident from the comparisons to a more accurate dietary method [42]. However, similarly low values for fruit and vegetable consumption were also found in some other Slovenian [4] and foreign [43] studies where fruit and vegetables were evaluated using other methods (FFQ or 3-day weighted dietary method).

#### Cereals/cereal products

The same applies to the consumption of cereals/cereal products. Only 32% of males and 25% of females met the OMD recommendations for cereals/cereal products, which has also been established by the results of other national [4, 18], and foreign studies [43, 17, 44, 6]. As consumption of fruits, vegetables, and whole grains importantly contributes to the maintenance of nutritional and energy balance and exerts protective effects against the development of chronic, non-communicable diseases, also reducing the risk of mortality, especially from cardiovascular diseases [45, 46], there obviously exists a long-term health risk in adolescents due to inadequate diet.

#### Sweet and salty snacks

In contrast, 71% of males and 82% of females in our sample exceeded the acceptable amount of sweet and salty snacks on average 3–4 times relative to OMD recommendation. Although no detailed analysis of the type of carbohydrate-containing foods consumed by adolescents was performed, the percentage of recommendations for cereals/cereal products and sweet and salty snacks suggests that the intake of whole grain products was too low in adolescents, whereas the opposite was true for the intake of processed or/and starchy foods. In addition, the study by Larson et al. [47] showed that consumption of high-energy salty snacks was associated with lower intake of fruits and vegetables and higher intake of sweet snacks, which is also a possible reason for the above-mentioned low intake of fruits and vegetables in the adolescents in our study. Consequently, reduced consumption of salty and sweet snacks could contribute to increased consumption of fruits and vegetables in adolescents.

#### Meat/meat products

Nearly three-quarters of adolescents consumed excessive amounts of meat/meat products (403% of the recommended value for males, 236% of the recommended value for females), which certainly increased the percentage of those adolescents, who met the recommendations for certain vitamins/minerals, particularly B vitamins such as niacin, for which most adolescents exceeded the recommended D-A-CH value by 50%. Selected meats, particularly liver and lean red and white meats, can contribute significantly to the intake of all B vitamins and vitamin A, and certain minerals such as iron, zinc, copper, molybdenum, fluoride and phosphorus [48]. Although detailed analyses in relation to different types of meat were not conducted in the present study, it can be assumed that adolescents consumed an excessive amount of different prepared meat products with added sodium chloride or sodium glutamate, as excessive sodium intake was evident in both genders (331% of the recommended value in females and 473% in males). This finding is also supported with study conducted by Partearroyo et al. [49]. Such high sodium intake can also be attributed to the increased consumption of salty snacks, as mentioned above.

#### Study limitations

First, similar to all other methods suitable for population studies, the 24-h recall for estimating dietary intake of vitamins/minerals has some drawbacks, yet this method was chosen by EFSA to collect data in national dietary studies for specific age groups of the population, including adolescents [22]. Therefore, due to standardization and harmonization of data between countries, which is one of the main objectives of EFSA, the choice of this method was deemed appropriate to assess the intake of vitamins/minerals in the population of Slovenian adolescents. Second, studies have revealed that, in addition to DS, the intake of fortified foods can also contribute significantly to increased intake of individual micronutrients [50]. Because data on the use of fortified foods have not been collected separately and the full range of fortified foods is not included in the OPEN database, intakes of some vitamins and/or minerals may have been somewhat underestimated. Moreover, although OPEN is a reliable tool for the assessment of macronutrients and selected essential minerals (Ca, Fe, Mg, Zn, Na, P, and Cu), the discrepancies between micronutrient assessment using OPEN and chemical measurements of specific nutrients such as iodine (-11%) and selenium (-19%) were quite large [25]. Furthermore, a number of validation studies have shown that non-reporting of ingested foods is the biggest problem in assessing nutrient intake in adolescents [33], which is also one of the more likely reasons for the low estimated intake of certain vitamins/minerals in our study. Last but not least, the available information from the product declarations of DS has been adopted as representative values for the vitamin/mineral intake from DS, although the possibility of discrepancies between the actual and the declared values are not excluded.

Considering that data for this study were collected in 2014, it is reasonable to assume, that the consumption of DS and/or fortified foods has in the meantime become more widespread among adolescents. Such and educated guess seems to be in accordance with the available literature [51, 52]. Consequently, micronutrient intake may not be as low, as the results of the present study suggest, yet, more recent data are needed to confirm it. Nevertheless, it is important to note that the potential risks associated with DS use may, for the same reason, become more concerning over time.

## Conclusion

Since the use of DS significantly reduces the percentage of adolescents with inadequate vitamin/mineral intake, one might conclude that the use of DS could be recommended from the perspective of short- and long-term adolescent health. However, we cannot agree with this view, as our data on the consumption of specific food groups reveal that adolescents do not consume sufficient amounts of most vitamins/minerals with diet alone to meet the recommended daily requirements for this age group. In addition, DS users have higher intakes of most vitamins/minerals from food alone than non-users, suggesting that DS is being taken by those adolescents, who actually need them less, further increasing the risk if DS are consumed in excess. We believe it is the utmost importance to promote adequate intake of specific food groups according to the existing recommendations, which in turn will lead to improved micronutrient intakes and reduce the need to use DS. Our study provided a new insight into the causes of inadequate intakes of individual vitamins/minerals, revealing serious inadequacies in adolescents’ intakes of food groups that we believe should be focused on. Instead of recommending vitamin/mineral supplementation in selected populations where deficiencies of individual vitamins/minerals exist, we should focus our activities on additional public health interventions to significantly improve the adolescents’ diets, particularly to achieve increased intakes of fruits, vegetables, cereals/cereal products, and milk/dairy products, and to reduce intakes of sweet and salty snacks and meat products, which will improve adequate micronutrient intakes in adolescents.

## Data Availability

The raw data supporting the conclusions of this article will be made available by the authors upon request to gregor.starc@fsp.uni-lj.si or gregor.jurak@fsp.uni-lj.si, without undue reservation.

## Abbreviations

ACDSi: The analysis of children’s development in Slovenia
BMI: Body mass index
D-A-CH: Reference values proposed by the nutrition societies of Germany, Austria, and Switzerland
DS: Dietary supplement
EFSA: European food safety authority
FFQ: Food frequency questionnaire
HuSKY: Healthy nutrition score for kids and youth
OMD: Optimized mixed diet
OPEN: Open platform for clinical nutrition
UL: Tolerable upper limit of daily intake
